# Immunity Elicited by an Experimental Vaccine Based on Recombinant Flagellin-Porcine Circovirus Type 2 Cap Fusion Protein in Piglets

**DOI:** 10.1371/journal.pone.0147432

**Published:** 2016-02-05

**Authors:** Shanshan Zhu, Chunyan Zhang, Jing Wang, Li Wei, Rong Quan, Jiayu Yang, Xu Yan, Zixuan Li, Ruiping She, Fengjiao Hu, Jue Liu

**Affiliations:** 1 Beijing Key Laboratory for Prevention and Control of Infectious Diseases in Livestock and Poultry, Institute of Animal Husbandry and Veterinary Medicine, Beijing Academy of Agriculture and Forestry Sciences, No. 9 Shuguang Garden Middle Road, Haidian District, Beijing, 100097, People’s Republic of China; 2 College of Veterinary Medicine, China Agricultural University, No. 2 Yuanmingyuan West Road, Haidian District, Beijing, 100197, People’s Republic of China; Federal University of Pelotas, BRAZIL

## Abstract

In a recent study, we reported that a recombinant protein from fusion expression of flagellin to porcine circovirus type 2 (PCV2) Cap induced robust humoral and cell-mediated immunity that afforded full protection for PCV2 infection using BALB/c mice. Here, we further evaluated the immunogenicity and protection of the recombinant protein using specific pathogen free (SPF) pigs. Twenty-five 3-week-old piglets without passively acquired immunity were divided into 5 groups. All piglets except negative controls were challenged with a virulent PCV2 at 21 days after booster vaccination and necropsied at 21 days post-challenge. Vaccination of piglets with the recombinant protein without adjuvant induced strong humoral and cellular immune responses as observed by high levels of PCV2-specific IgG antibodies and neutralizing antibodies, as well as frequencies of PCV2-specific IFN-γ-secreting cells that conferred good protection against PCV2 challenge, with significant reduced PCV2 viremia, mild lesions, low PCV2 antigen-positive cells, as well as improved body weight gain, comparable to piglets vaccinated with a commercial PCV2 subunit vaccine. These results further demonstrated that the recombinant flagellin-Cap fusion protein is capable of inducing solid protective humoral and cellular immunity when administered to pigs, thereby becoming an effective PCV2 vaccine candidate for control of PCV2 infection.

## Introduction

Porcine circovirus type 2 (PCV2)-associated diseases (PCVAD) [1–3] are known to include varieties of clinical syndromes, such as postweaning multisystemic wasting syndrome (PMWS), porcine respiratory disease complex, reproductive failure, dermatopathy and nephropathy syndrome, and growth retardation. In spite of improved management strategies for control of PCVAD, vaccination is still used cost-effectively for preventing pigs from PCV2 infection and several kinds of vaccines have been shown to be effective in controlling PCV2-associated diseases [4,5]. Besides inactivated PCV2 vaccines derived from conventional PCV2- or genetically engineered chimeric PCV1-2-infected PK15 cells, *there are* several kinds of PCV2 subunit vaccines have been developed from the expression of PCV2 ORF2-encoded protein (Cap), which is thought to be the host-protective immunogen [6,7]. These PCV2 vaccines included bacterial-vectored vaccines [8], baculovirus-vectored vaccines [9], yeast-vectored vaccine [10], live-vectored expression vaccines [11–13], and DNA vaccines [14,15]. Of these vaccines, three kinds of PCV2 subunit vaccines, including baculovirus-expressed recombinant Cap protein [4,5], have been used for pig vaccination against PCV2 infection. These commercial PCV2 subunit vaccines used in pig vaccination regime have been shown are able significantly to reduce culling rates and promote growth performance in PCVAD-stricken farms, thereby alleviating the economic losses caused by PCV2 infection-associated diseases.

In general, induction of a solid humoral response was used as an important parameter for evaluation of immune efficacy of commercial PCV2 vaccines; however, increased research data suggested that the interaction between PCV2 infection and immune system including humoral and cellular immune responses plays a major role in the PMWS pathogenesis [16–20]. High PCV2 viremia contribute to PMWS occurrence [21,22]. Reduced PCV2 viremia has been shown to correspond to the production of both neutralizing antibodies and interferon-γ response during PCV2 infection in pigs [21–24]. Therefore, the production of interferon-γ-secreting cells, as measurable for cellular immune response, is also another important parameter to evaluating the protective immunity of commercial PCV2 vaccines against PCV2 infection.

In a recent work [25], we evaluated the immunogenicity and protection of a recombinant flagellin-Cap protein using baculovirus expression in BALB/c mice. The results of that study showed that the recombinant flagellin-Cap protein could confer better humoral and cell-mediated immune responses for full protecting mice against PCV2 infection than those in the recombinant Cap alone-inoculated mice, thereby serving as an attractively alternative vaccine for controlling PCV2-associated diseases. In this study, we continue to determine whether the immunity induced by the recombinant protein was efficient enough for protecting piglets from PCV2 infection. For that, the immunogenicity and protection of the recombinant protein was evaluated in terms of induction of humoral and cellular immunity, and PCV2 infection by measuring PCV2 viral DNA loads in sera, microscopic lesions and viral antigen positive signals in lymph node tissues.

## Materials and Methods

### Ethics Statement

This study was performed in line with the animal welfare guidelines of the Institutional Animal Care and Use Committee (IACUC) under the approval of Institute of Animal Husbandry and Veterinary Medicine which is affiliated to Beijing Academy of Agriculture and Forestry Sciences (Permit number: 2014–05). All efforts were made to alleviate animal suffering.

### Expression and purification of recombinant flagellin-Cap fusion protein

Sf9 cells were infected with a recombinant baculovirus expressing flagellin-PCV2 Cap fusion protein [25] and harvested at 72 h post-infection, and the recombinant protein was purified as described by Zhang et al. [25].

### Immunization of piglets

A total of 25 30-day-old, specific-pathogen–free (SPF) Duroc × Large-White male piglets, from Beijing Center for SPF Swine Breeding & Management, China, were randomly allocated in 5 groups (5 piglets of each group) and housed in isolation rooms and fed with sterilied food as well as water ad libitum. The absence of PCV2 viremia in the 25 piglets was confirmed by polymerase chain reaction (PCR) detection. Piglets from groups 1 and 2 were immunized intramuscularly in neck with 200 μg (200rFla-Cap) and 500 μg (500rFla-Cap) of the recombinant flagellin-Cap protein without any adjuvant, respectively. Group 3 was immunized with a commercial PCV2 subunit vaccine (Ingelvac^®^ CIRCOFLEX™, Böehringer Ingelheim) (Subunit-Vac) at a dose according to the manufacturer’s protocol. Groups 4 and 5, which were inoculated with sterile phosphate-buffered saline (PBS), served as non-vaccinated PCV2-challenged (NV-C) and non-vaccinated non-challenged (NV-NC) group, respectively. Each vaccinated group was boosted three weeks later. All of the piglets from groups 1 to 4 were inoculated intranasally with 2 ml of 10^5^ TCID_50_/ml PCV2 strain BJW [26] at 21 days after booster. All of the pigs were monitored daily for the presence of varieties of clinical signs. Rectal temperature was measured for three consecutive days for all of the pigs at the first week after PCV2 challenge. Body weight was recorded from all pigs at primary and booster immunization, and at challenge and at 21 days after challenge. Blood samples at 2 to 3 ml of volume for each pig were collected from the anterior vena cava at weekly intervals following primary and booster vaccination, and at 21 days following PCV2 challenge. At 21 days after challenge, serum samples from all the group pigs were taken for determining PCV2 viral DNA loads, and all of the pigs were humanely euthanized after intravenous injection of 80 mg/kg body weight sodium pentobarbital, and a complete necropsy was performed. In addition, inguinal lymph nodes from all of the group pigs were collected for histopathological analysis. Animal research was reported for this study following the ARRIVE Guidelines [27]. An entire ARRIVE guidelines checklist is provided in S1 ARRIVE Checklist.

### Quantification of PCV2 viremia by qPCR

Quantitative real-time PCR (qPCR) was used for determining PCV2 viral DNA loads in serum samples as described by Zhang et al. [25].

### Serology

The serum samples from pigs at the indicated times were determined for antibodies against Cap protein with an indirect enzyme-linked immunosorbent assay (ELISA) by using recombinant Cap protein as a coating antigen and for PCV2 neutralizing antibodies (NA) with an end-point dilution reduction assay as described by Zhang et al. [25].

### Enzyme-linked immunospot (ELISPOT) assay

For the assessment of cell-mediated immunity, the frequencies of PCV2-induced interferon-γ-secreting cells (IFN-γ-SC) from porcine peripheral blood mononuclear cells (PBMC) at the indicated times were determined using a commercial ELISPOT assay kit (Mabtech) in accordance with the manufacturer’s instructions with minor modifications. In brief, whole blood samples were heparinized and PBMC were isolated following density gradient centrifugation in Histopaque-1007 (Sigma) and plated at 2×10^6^ cells/well in RPMI-1640 medium containing 10% fetal bovine serum (FBS) into 96-well Plates pre-coated with anti-porcine IFN-γ monoclonal antibody (10 μg/ml, Mabtech), and stimulated with 2 μg/ml of purified recombinant PCV2 Cap protein. The plates were subsequently incubated with 0.5 μg/ml anti-swine IFN-γ biotin-labeled mAb at 37°C for 2 h, followed by incubation with streptavidin-peroxidase diluted 1:100 in 0.5% FBS + PBS at 37°C for 1 h and then incubated for 30 min with an AEC solution for 30 min. This reaction was finally stopped with distilled water. In addition, phytohemagglutinin (10 μg/ml) and RPMI medium served as positive and negative controls, respectively. The frequencies of PCV2-specific IFN-γ-SC were obtained by subtracting the mean spot numbers of the negative control wells from those observed upon stimulation with Cap protein and expressed as the frequencies of the responding cells for per million PBMC. Samples with 20 or over specific spots served as positive for Cap-specific IFN-γ response as described elsewhere [28].

### Histopathology and immunohistochemistry

The inguinal lymph nodes from all the group pigs collected at 21 days after PCV2 challenge were formalin-fixed, paraffin-embedded, sectioned, and stained with haematoxylin/eosin (HE) for microscopic examination. The lymph node tissues for histopathological observation were subjected to immunohistochemistry (IHC) staining for detection of PCV2 viral antigen. The scores of histopathological lesions [19] were ranged from 0 (normal) to 3 (severe) for observing lymphoid depletion, infiltration of histiocytes, histiocytic hyperplasia, syncytial giant cell formation, or granulomatous replacement. A rabbit polyclonal antibody against Cap protein was used for IHC as described previously [29]. The scores of PCV2 antigen were ranged from 0 for no signal to 3 for strong positive signals.

### Statistical analysis

Results are presented as means ± the standard deviation (SD) of the means, as indicated. One-way analysis of variance (ANOVA) and Student’s *t* test were used for statistical analysis, and P<0.05 was considered to be statistically significant differences between groups.

## Results

### Clinical presentation

The clinical signs of PMWS mainly include fever, emaciation, dyspnea, paleness of the skin, and, less frequently, jaundice and diarrhea. None of the non-vaccinated non-challenged and vaccinated PCV2-challenged pigs exhibited clinical signs or remarkable macroscopic lesions compatible with PMWS for the duration of the experiment. However, all of the non-vaccinated PCV2-challenged pigs (5/5) exhibited *≥* 40.0°C (ranging from 40.0 to 40.3°C) of rectal temperatures in three consecutive days at the first week after PCV2 challenge, but returned to normal levels thereafter. Furthermore, three out of five non-vaccinated PCV2-challenged pigs exhibited lung lesions characterized by small, focal, solid areas of pneumonia.

For mean body weight, no statistical differences were observed within the groups at all the indicated time points after vaccination (data not shown). However, the average body weights of all of the vaccinated pigs regardless of vaccination of rFla-Cap or Subunit-Vac were higher (p<0.05) than that of non-vaccinated pigs after PCV2 challenge (Table 1). Vaccinated pigs had an increased average daily weight gain (ADWG) of 52, 65, and 49 g/day in the 200rFla-Cap, 500rFla-Cap, and Subunit-Vac groups, respectively, as compared to non-vaccinated pigs during the PCV2 challenge (Table 1). In addition, the non-vaccinated pigs exhibited the maximum body weight loss, with an average body weight loss being 2.36 kg, as compared to the 500rFla-Cap-vaccinated pigs during the PCV2 challenge.

**Table 1:** Average body weights of rFla-Cap vaccinated pigs following PCV2 challenge.

Group	Body weight (kg)^a^		Average daily weight gain (kg)
	At challenge	21 days after challenge	
200rFla-Cap	25.45 ± 1.92	42.94 ± 2.76*	0.833 ± 0.265
500rFla-Cap	26.32 ± 1.75	44.09 ± 1.98*	0.846 ± 0.216
Subunit-Vac	25.98 ± 2.13	43.41 ± 2.35*	0.830 ± 0.238
NV-C	25.32 ± 2.26	41.73 ± 2.69	0.781 ± 0.271
NV-NC	25.87 ± 2.95	42.81 ± 2.95	0.807 ± 0.313

^a^ Asterisks (*) within columns represent significantly difference of average body weights between the rFla-Cap- or Subunit-Vac-vaccinated and non-vaccinated PCV2-challenged pigs after challenge (*P*<0.05).

### Humoral immune response to PCV2

**Fig 1:**
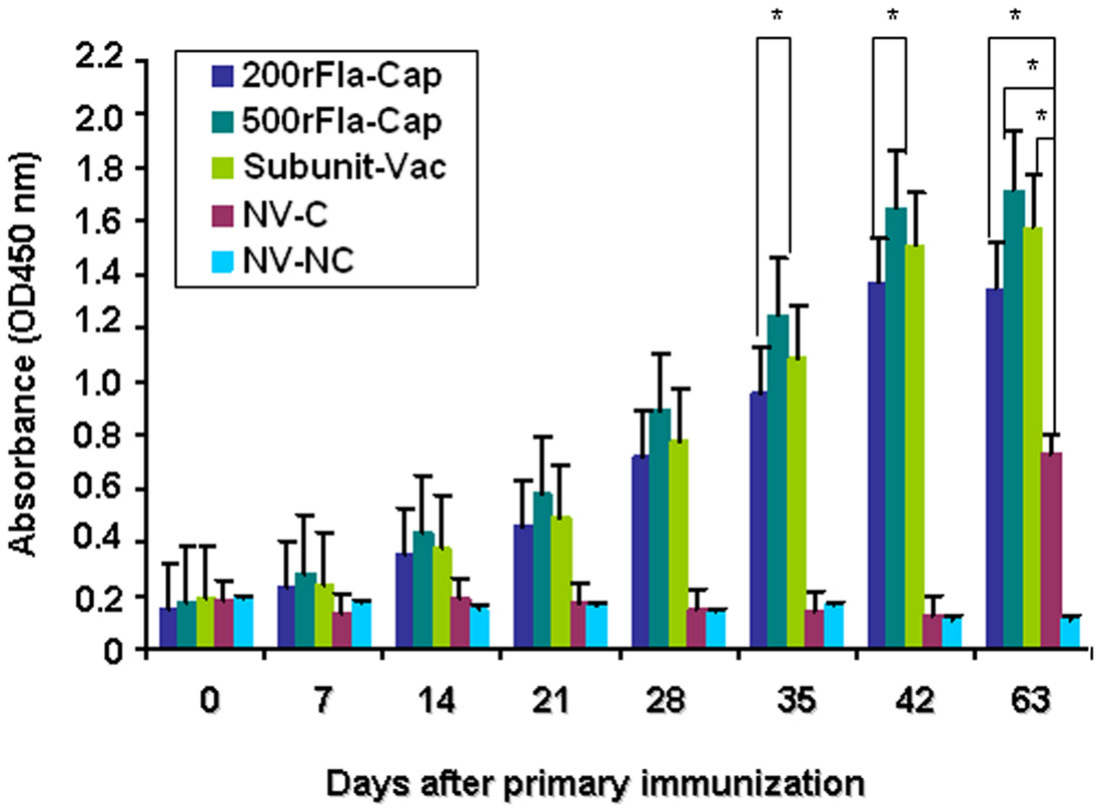
Kinetics of mean PCV2 Cap-specific antibodies in pigs vaccinated with rFla-Cap protein after vaccination and PCV2 challenge using an ELISA method. Results indicate mean absorbance values of serum samples for each group ± SD from one of two independent experiments. *p*<0.05 (*) represents significant difference between 500rFla-Cap and 200rFla-Cap-vaccinated pigs at the indicated times after vaccination, or vaccinated and non-vaccinated pigs after PCV2 challenge (day 63).

An indirect ELISA was used to measure the titers of serum antibodies raised against Cap protein in the vaccinated piglets. As shown in Table 2, all of the non- vaccinated and non-challenged piglets were negative for anti-Cap specific antibody for the whole duration of the experiment. At the time of vaccination, the piglets in all the 5 groups were seronegative against Cap-specific antibody. The dynamics of PCV2 Cap-specific antibodies for the duration of the experiment are displayed in Fig 1. The Cap-specific antibody titers were significantly higher (p<0.05) in the pigs vaccinated with 500rFla-Cap than in the pigs vaccinated with 200rFla-Cap at 35 and 42 days post-vaccination (dpv). By comparison with the piglets vaccinated with Subunit-Vac, the 500rFla-Cap-vaccinated piglets had higher mean PCV2 antibody levels after vaccination, while the piglets vaccinated with 200rFla-Cap exhibited lower mean PCV2 antibody levels after vaccination. However, there was no significantly difference between the mean Cap-specific antibody titers of the Subunit-Vac- and 500rFla-Cap- or 200rFla-Cap-vaccinated piglets from 7 to 28 dpv. After PCV2 challenge, the Cap protein-specific antibody levels found in the two rFla-Cap-vaccinated groups and Subunit-Vac-vaccinated group remained significantly higher (p<0.05) than those found in the non-vaccinated PCV2-challenged group piglets.

**Table 2:** Seroconversion to PCV2 Cap-specific antibodies in pigs vaccinated with rFla-Cap proteins.

Group	No. of pigs with Cap antibodies/no. tested^a^ at days after vaccination						
	7	14	21	28	35	42 (challenge)	63^b^
200rFla-Cap	0/5	1/5	3/5	5/5	5/5	5/5	5/5
500rFla-Cap	0/5	3/5	5/5	5/5	5/5	5/5	5/5
Subunit-Vac	0/5	1/5	4/5	5/5	5/5	5/5	5/5
NV-C	0/5	0/5	0/5	0/5	0/5	0/5	5/5
NV-NC	0/5	0/5	0/5	0/5	0/5	0/5	0/5

^a^ Sera of all the five pigs from each group were taken at the indicated times after vaccination.

^b^ Sera of all the five pigs from each group were taken at 21 days after PCV2 challenge.

**Fig 2:**
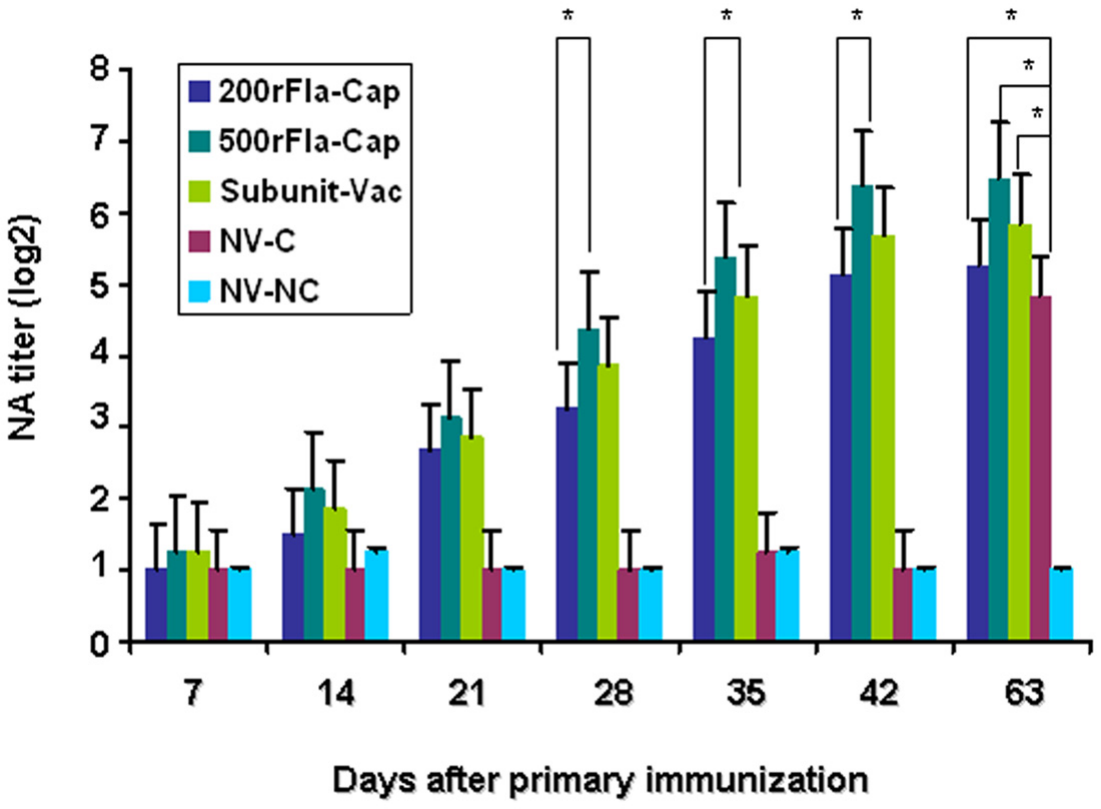
PCV2 neutralizing antibody responses in pigs vaccinated with rFla-Cap protein after vaccination and PCV2 challenge by using an end-point dilution reduction assay. Results indicate mean antibody titers of serum samples for each group ± SD from one of two independent experiments. *p*<0.05 (*) represents significant difference between 500rFla-Cap and 200rFla-Cap-vaccinated pigs at the indicated times after vaccination, or vaccinated and non-vaccinated pigs after PCV2 challenge (day 63).

We continued to determine PCV2 specific neutralizing antibody (NA) titers of sera of vaccinated pigs collected from the indicated times following vaccination and challenge. At the time of vaccination, no detectable neutralizing antibodies were seen in all of the five group piglets (data not shown). As dispayed in Fig 2, PCV2 serum NA titer in 1 out of 5 piglets in group 1 (200rFla-Cap) was positive at 14 dpv and steadily increased thereafter, with a titer of 1:38.4 at 42 dpv. In group 2 (500rFla-Cap), the NA titers of 3 out of 5 piglets were positive at 14 dpv and then increased significantly thereafter, with a titer of 1: 89.6 at 42 dpv. In group 3 (Subunit-Vac), the NA titers of 2 out of 5 piglets were positive at 14 dpv and steadily increased thereafter, with a titer of 1:57.6 at 42 dpv. The NA titers were significantly higher (p<0.05) in the animals vaccinated with 500rFla-Cap than in the animals vaccinated with 200rFla-Cap at 28 to 42 dpv. After PCV2 challenge, the NA titers were significantly higher (p<0.05) in the two rFla-Cap-vaccinated groups and Subunit-Vac-vaccinated group than that in the non- vaccinated PCV2-challenged group. No detectable specific neutralizing antibodies against PCV2 were observed in the non-vaccinated non-challenged pigs throughout the experiment.

These results demonstrated clearly that the recombinant flagellin-Cap protein, without addition of any adjuvant, was capable of eliciting strong humoral immune response as evidenced by high serum PCV2-specific antibody and neutralizing antibody levels comparable to the commercial PCV2 Cap subunit vaccine in piglets.

### PCV2-specific interferon-γ-secreting cells

**Fig 3:**
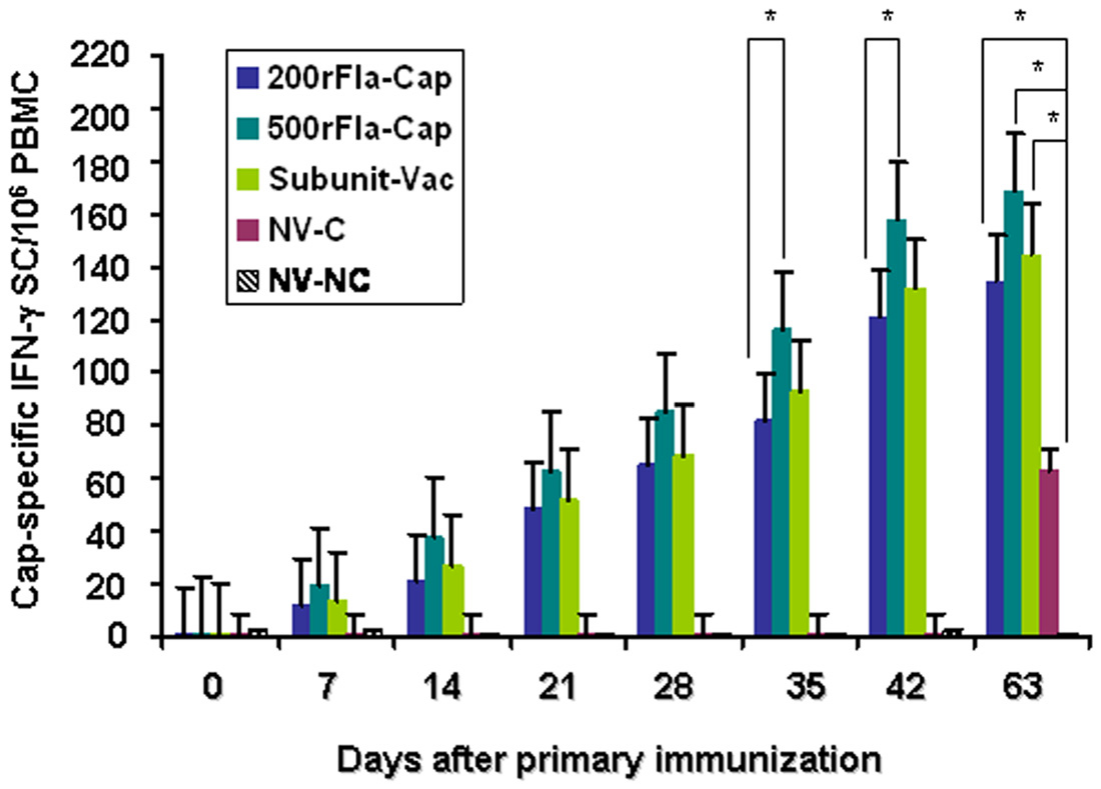
Mean frequencies of Cap-specific interferon-γ-secreting cells (IFN-γ-SC) per million of PBMC were determined after vaccination and PCV2 challenge. Results indicate mean Cap-specific IFN-γ-SC frequencies of each group samples ± SD from one of two independent experiments. *p*<0.05 (*) represents significant difference between 500rFla-Cap and 200rFla-Cap-vaccinated pigs at the indicated times after vaccination, or vaccinated and non-vaccinated pigs after PCV2 challenge (day 63).

In the recent report [25], we determined the cytokines TNF-α and IFN-γ levels to assess cellular responses elicited by the flagellin-Cap protein in the mouse model. Therefore, we here determined Cap-specific IFN-γ-SC in PBMC after immunization with the rFla-Cap protein in the pig experiment. Induction of Cap-specific IFN-γ -SC from each group was displayed in Fig 3. At the time of vaccination, the frequencies of Cap-specific IFN-γ-SC were negligible (0-3/10^6^ PMBC) in all of the 5 group piglets. In group 1, 2/5 piglets responded to Cap protein, possessing average frequencies of Cap-specific IFN-γ-SC of 21 ± 6/10^6^ PMBC at 14 dpv and increased steadily by 42 dpv (5/5 piglets with an average frequency of Cap-specific IFN-γ-SC of 121 ± 16/10^6^ PMBC). For group 2 piglets, induction of Cap-specific IFN-γ-SC appeared at day 14 dpv (4/5 piglets with an average frequency of Cap-specific IFN-γ-SC), and increased statistically thereafter, with an average frequency of Cap-specific IFN-γ-SC of 158 ± 21/10^6^ PMBC at 42 dpv. For group 3 piglets, 3/5 pigs responded to Cap protein possessed an average frequency of 27 ± 8/106 PMBC IFN-γ-SC at 14 dpv, and increased steadily by an average frequency of 132 ± 19/10^6^ PMBC Cap-IFN-γ-SC at 42 dpv. The average frequencies of Cap-specific IFN-γ-SC were significantly higher (p<0.05) in the animals vaccinated with 500rFla-Cap than in the animals vaccinated with 200rFla-Cap at 35 and 42 dpv. After PCV2 challenge, the average frequencies of Cap-specific IFN-γ-SC were significantly increased (p<0.05) in the two rFla-Cap- and Subunit-Vac-vaccinated groups in comparison with the non-vaccinated PCV2-challenged pigs. The frequencies of Cap-specific IFN-γ-SC were negligible (0-3/10^6^ PBMC) in the non-vaccinated non-challenged pigs throughout the experiment.

### PCV2 DNA loads in sera

We determined serum PCV2 viral loads of all of the group pigs after PCV2 challenge using real- time quantitative PCR. As displayed in Table 3, all of the non- vaccinated PCV2-challenged pigs exhibited PCV2 viraemia with the mean genomic copy numbers of 8.5 ± 1.2 log_10_ PCV2 copies/ml. As compared to the non-vaccinated PCV2-challenged control, PCV2 viremia was detected for two out of five pigs in the 200rFla-Cap vaccinated group, none of five pigs in the 500rFla-Cap vaccinated group, and only one out of five pigs in the Subunit-Vac vaccinated group. Besides all the pigs in the 500rFla-Cap vaccinated group remained negative for PCV2 viraemia, the two other groups showed significant decreases in the levels of viral DNA loads (P<0.05) when compared to the non-vaccinated PCV2-challenged group, with the mean genomic copy numbers of 2.3 ± 0.6 and 2.1 log_10_ PCV2 copies/ml for the 200rFla-Cap- and Subunit-Vac-vaccinated groups, respectively. No PCV2 viral DNA of the serum samples was detected from the non-vaccinated non-challenged pigs.

**Table 3:** Viral DNA load in the serum samples of rFla-Cap vaccinated pigs following PCV2 challenge.

Group	No. of pigs with viremia/no. tested (mean log PCV2 load ± SD) after challenge^a^
200rFla-Cap	2/5 (2.3 ± 0.6) *
500rFla-Cap	0/5 (0)
Subunit-Vac	1/5 (2.1)*
NV-C	5/5 (6.8 ± 1.6)
NV-NC	0/6 (0)

^a^ Asterisks (*) within columns represent significantly difference of viral DNA loads between the rFla-Cap- or Subunit-Vac-vaccinated and non-vaccinated PCV2-challenged pigs after challenge (*P*<0.05).

### Microscopic lesions and IHC staining

**Fig 4:**
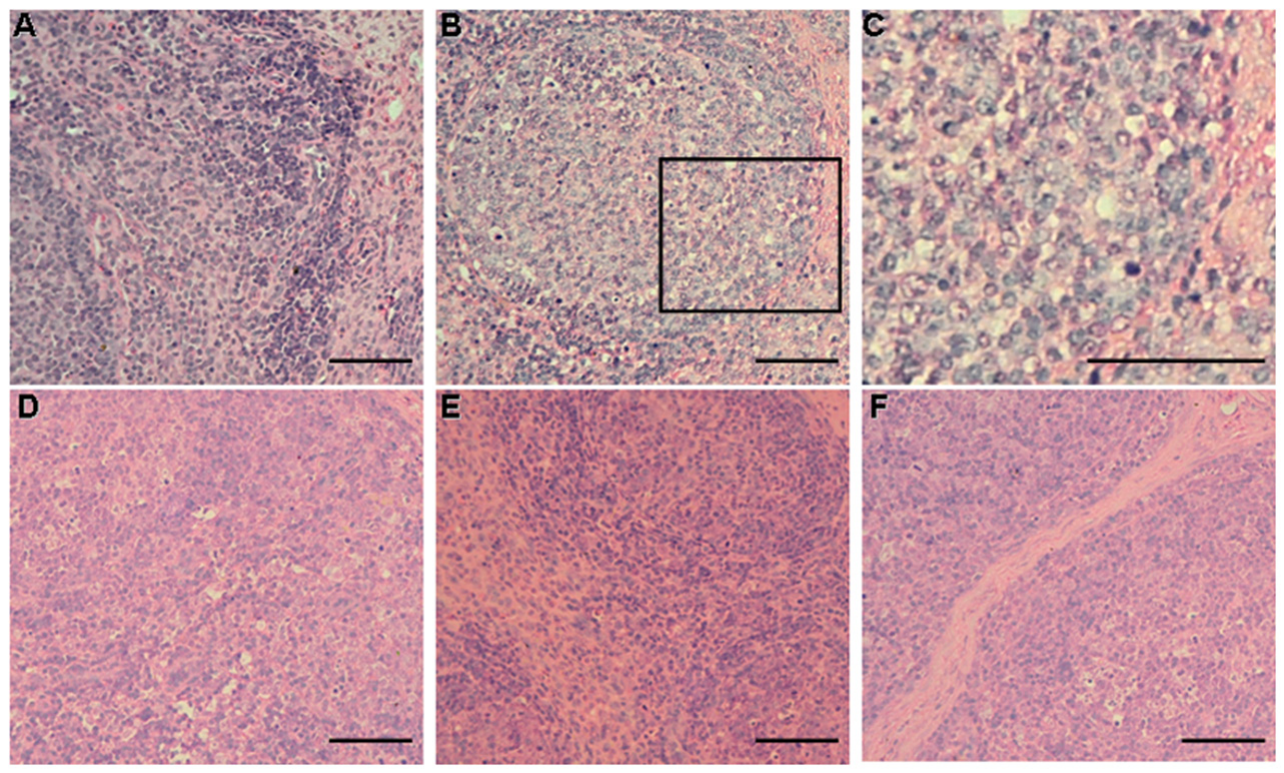
Histopathologic changes in the inguinal lymph nodes of rFla-Cap vaccinated pigs after PCV2 challenge. (A) Inguinal lymph node section from a non-vaccinated non-challenged pig shows no noticeable microscopic lesions. (B) Severe microscopic lesions of lymph nodes from a non-vaccinated PCV2-challenged pig. Depletion of lymphocytes, infiltration of histiocytes, and syncytial cells were seen. (C) Enlargement of some areas (black square) in panel B. No or mild microscopic lesions were seen in the lymph node of a 200rFla-Cap-vaccinated PCV2-challenged (D) or Sub-Vac-vaccinated PCV2-challenged (F) pig. (E) No noticeable lesions were seen in the lymph node of a 500rFla-Cap-vaccinated PCV2-challenged pig. Bar, 80 μm.

To further evaluate the protective immunity of the rFlan-Cap protein, the inguinal lymph nodes were taken from all the group pigs at 21 dpc for histopathological observation and PCV2 antigen detection. As shown in Fig 4A, no obvious histopathological changes were observed in the non-vaccinated non-challenged pigs after PCV2 challenge. In the non-vaccinated PCV2-challenged group, all of the pigs had microscopic lesions, including diffuse infiltration of histiocytes, lymphoid depletion and hyperplasia, germinal center development, or syncytial giant cell formation (Fig 4B and data not shown). However, no or mild lymphoid damage and syncytial giant cell formation (Fig 4D and 4E) were observed for the rFla-Cap-vaccinated PCV2-challenged pigs, with the 500rFla-Cap-vaccinated group pigs showing no obvious microscopic lesions. No or mild lymphoid depletion, hyperplasia, or a few amounts of syncytial giant cells were seen in the Subunit-Vac-vaccinated PCV2-challenged pigs (Fig 4F). Mean scores of microscopic lesions for inguinal lymph nodes were statistically lower (p<0.05) in the vaccinated PCV2-challenged pigs than in the non-vaccinated PCV2-challenged pigs (Table 4). Moreover, the mean scores of histopathological changes in those pigs from the vaccinated PCV2-challenged groups were not statistically different one another (Table 4).

**Table 4:** Histopathological lesion scores for inguinal lymph nodes of rFla-Cap vaccinated pigs after PCV2 challenge.

Group	Mean lesion scores^a^
200rFla-Cap	0.8 ± 0.45*
500rFla-Cap	0.2 ± 0.45*
Subunit-Vac	0.6 ± 0.55*
NV-C	2.6 ± 0.55
NV-NC	0

^a^ Asterisks (*) within columns represent significantly difference of mean lesion scores between the rFla-Cap- or Subunit-Vac-vaccinated and non-vaccinated PCV2-challenged pigs after challenge (*P*<0.05). The data show the mean scores of five pigs in each group from one of two independent experiments.

**Fig 5:**
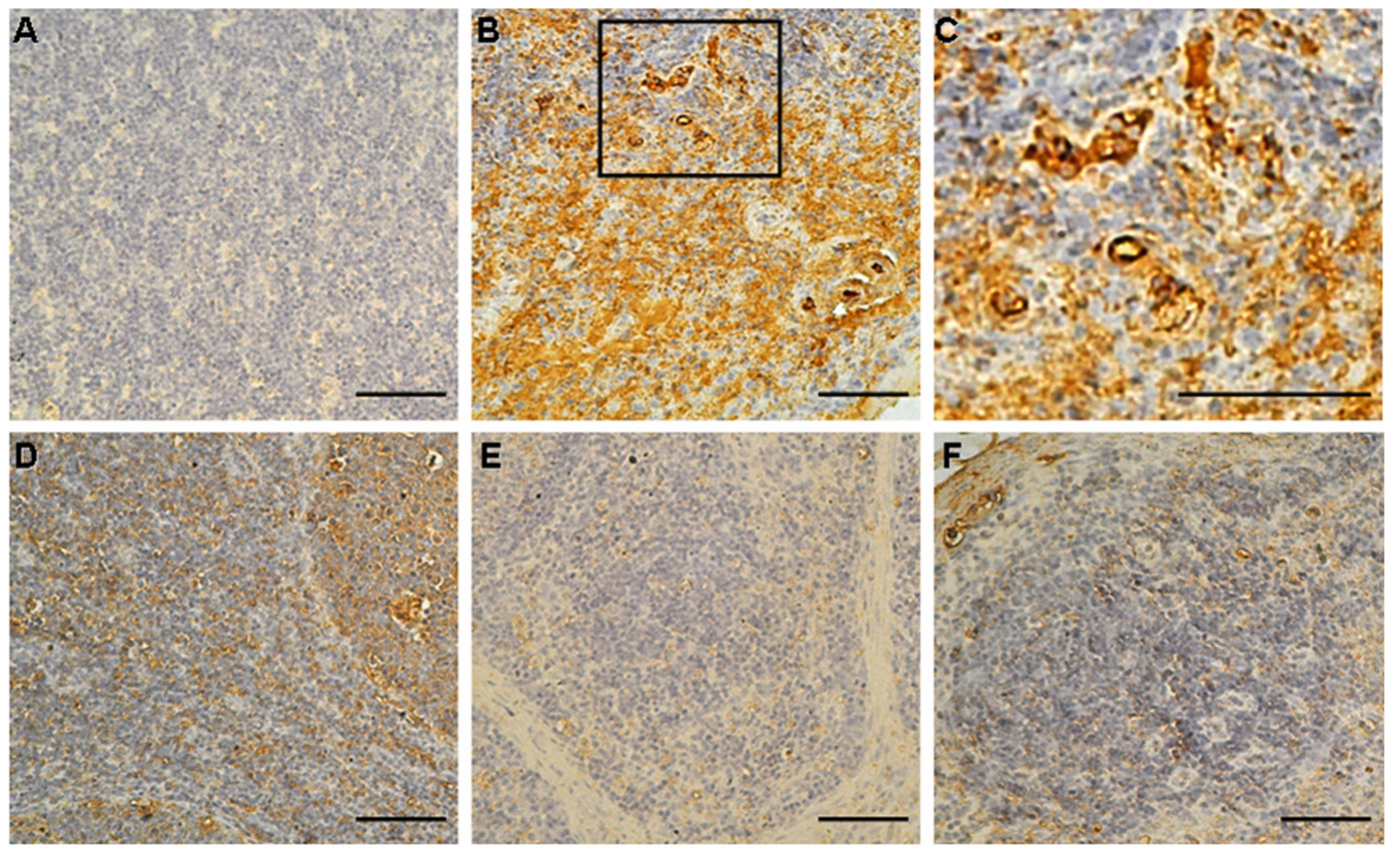
Immunohistochemical staining for inguinal lymph nodes of rFla-Cap vaccinated pigs after PCV2 challenge. Cells were positive for PCV2 viral antigen (brown). (A) Lymph node section from a pig of the non-vaccinated non-challenged group shows no staining for PCV2 antigen. (B) Lymph node section from a non-vaccinated PCV2-challenged pig. Many PCV2 antigen-positive cells are seen. (C) Enlargement of some areas (black square) in panel B. (D) Lymph node section from a 200rFla-Cap-vaccinated PCV2-challenged pig shows reduced amounts of PCV2-positive cells. (E) 500rFla-Cap-vaccinated PCV2-challenged pig. No staining for PCV2 viral antigen is seen. (F) Sub-Vac-vaccinated PCV2-challenged pig. A small amount of positive cells for PCV2 viral antigen are seen. Bar, 80 μm.

The inguinal lymph nodes taken at 21 days after PCV2 challenge were further subjected to detecting PCV2 viral antigen by IHC analysis. As expected, all of the lymph nodes from the non-vaccinated non-challenged group pigs were absent for PCV2 antigen. Within the rFla-Cap-vaccinated groups, low to medium levels of PCV2 viral antigen were seen in 2 out of 5 pigs vaccinated with 200rFla-Cap but not detected in any of the 5 pigs vaccinated with 500rFla-Cap. For Subunit-Vac-vaccinated group, low levels of viral antigen were seen in the inguinal lymph nodes of 2 out of 5 pigs after PCV2 challenge. In the non-vaccinated PCV2-challenged group, medium to severe levels of viral antigen were seen in the inguinal lymph nodes of all the 5 pigs. As shown in Fig 5, the PCV2-positive cells were mainly macrophages and histiocytic cells, but lymphocytes were also stained positive for PCV2 antigen. In addition, weak staining was observed frequently in syncytia. The mean scores for the levels of PCV2 viral antigen in the vaccinated PCV2-challenged animals were significantly lower than (p<0.05) in non-vaccinated PCV2-hallenged animals (Table 5). Within the vaccinated groups, the 500rFla-Cap vaccinated pigs exhibited the lowest PCV2 antigen amounts, whilst the 200rFla-Cap vaccinated pigs showed the highest PCV2 antigen amounts, but comparable to that in the Subunit-Vac vaccinated pigs (Table 5).

**Table 5:** PCV2 antigen scores for inguinal lymph nodes of rFl-Cap vaccinated pigs after PCV2 challenge by immunohistochemical detection.

Group	Mean PCV2 antigen scores^a^
200rFla-Cap	1.2 ± 0.45*
500rFla-Cap	0.2 ± 0.45*
Subunit-Vac	0.8 ± 0.45*
NV-C	2.8 ± 0.45
NV-NC	0

^a^ Asterisks (*) within columns represent significantly difference of mean viral antigen scores between the rFla-Cap- or Subunit-Vac-vaccinated and non-vaccinated PCV2-challenged pigs after challenge (*P*<0.05). The data show the mean scores of five pigs in each group from one of two independent experiments.

## Discussion

PCVAD serves as a major infectious disease occurred in many pig-rearing countries, causing severely economic losses in the pig industry around the world. A number of PCV2 vaccines, based upon development of inactivated PCV2 and expression of Cap protein, have been used to vaccinate pigs against PCV2 infection and shown to be a cost-effective strategy for controlling PCVAD occurrence. In our recent study, we reported that a recombinant flagellin-Cap protein based on baculovirus expression system was capable of eliciting a strong immune response that conferred full protection for mice from PCV2 infection. In this study, we further used the recombinant flagellin-Cap protein to immunize piglets for evaluating its immunogenicity and protective capabilities against PCV2 infection, in comparison with an availably commercial Cap subunit vaccine. Our results clearly showed that piglets vaccinated with the flagellin-Cap protein without addition of any adjuvant induced strong humoral and cellular immune responses, low levels of virimea, as well as mild microscopic lesions and less PCV2-positive cells that confer good protection for pigs from PCV2 challenge, comparable to those pigs in the commercial Cap subunit vaccine.

Increasing research data have suggested that SPF pigs or PCV2 antibody free pigs have been used to evaluate the efficacies of PCV2 vaccines [2,14,30]. Although PCV2 acts as the major etiological agent for PMWS, a number of co-factors or triggers are considered to be required for the occurrence of the disease. This leads usually to the failure of reproducing PMWS in the pig experimental model using infection of PCV2 alone. However, as performed in previous studies [4,31–33], the efficacy of PCV2 vaccines may be evaluated using some parameters related to PCV2 infecton, including viremia burden, viral-induced specific lymphoid lesions, viral loads in tissues, as well as development of humoral and cellular immune responses. In the present study, we found that the piglets did not appear any clinical disease compatible with PMWS when challenged with PCV2 alone; however, we found that all of the non-vaccinated PCV2-challenged pigs exhibited viremia. In addition, moderate to severe microscopic lesions and medium to high amounts of PCV2 viral antigens were also seen in the inguinal lymph nodes of the non-vaccinated PCV2-challenged pigs. All of the 500rFla-Cap vaccinated animals were fully protected from PCV2 viremia, while 2 out of 5 pigs in the 200rFla-Cap vaccinated group appeared PCV2 viremia; however, in those two pigs with viremia, PCV2 viral DNA levels were significantly decreased compared to the non-vaccinated challenged pigs. The mean scores of the histopathological changes for the inguinal lymph nodes of the rFla-Cap vaccinated groups showed that the severity of lesions were significantly lower (p<0.05) than those of the non-vaccinated group after PCV2 challenge. Moreover, PCV2-positive antigen signals on lymph nodes exhibited differences between vaccinated and non-vaccinated PCV2-challenged pigs; the mean score of PCV2-positive antigen signals in the lymph nodes of the non-vaccinated PCV2-challenged pigs was significantly higher than (p<0.05) those in rFla-Cap-vaccinated PCV2-challenged pigs after challenge. These results indicated that the rFla-Cap protein was capable efficiently of reducing viremia burden, viral-induced specific lymphoid lesions, and PCV2-positive cells in lymph nodes, comparable to commercial PCV2 subunit vaccine.

PCV2-specifc antibodies have been shown to be associated with protection because inefficient or delayed induction of humoral immunity to PCV2 contributes to high levels of PCV2 viral loads and the development of PCVAD [22,23,34–36]. In this study, the PCV2-specific antibodies assayed by ELISA revealed that the antibody levels of the serum samples from the rFla-Cap vaccinated pigs gradually increased in a dose-dependent manner after vaccination. Vaccination of pigs with rFla-Cap protein also leads to increased titers of neutralization antibody, which afforded good protection against PCV2 challenge, with kinetics that paralleled those observed for sera antibodies determined by ELISA. These results further confirmed that the rFla-Cap protein without addition of any adjuvant could induce a high level of antibody-mediated immune response comparable to that in the commercial subunit Cap vaccine in swine. Increasing research evidence have suggested that specific cellular response play a role in the protection against PMWS [28,32,37]. IFN-γ, a key Th1 cytokine with immunomodulatory effects, mediates cellular immunity against virus infections by controlling the differentiation of naïve CD4^+^ cells for production of CD4^+^ cells. In this study, we determined the induction of cell-mediated immunity in response to rFla-Cap protein vaccination in piglets and found that vaccination of the rFla-Cap protein elicites a specific cellular immune response with a statistical increase of the frequency of IFN-γ-SC from 14 days thereafter. We also found that those pigs that were PCV2 viral antigen-positive and had high levels of viral DNA loads, corresponded to those possessing the lower frequency of IFN-γ-SC before PCV2 infection. These results are consistent with that reported by Fort et al. [32] and Martelli et al. [38], cell-mediated response, which is measurable as IFN-γ-SC, could attribute, together with PCV2 neutralizing antibodies, to clearing virus and blocking progression of infection.

Flagellin, an agonist of Toll-like receptor 5 (TLR5), has been used as an adjuvant for varieties of model-system vaccinations [39,40]; thereby stimulating innate immune responses followed by establishing humoral and cellular immunity [41–43]. When flagellin fused to bacterial or viral proteins, inculding Yersinia pestis [44], West Nile virus [45], poxvirus [46], and influenza virus [47], it was demonstrated enhancement of the immunization efficacy as evidenced by stronger humoral and cellular immune responses. In a mouse experimental model [25], we found that the flagellin fused to PCV2 Cap protein promoted higher production of antibody levels and higher upregulation of IFN-γ, as compared to the Cap protein alone. These results were further confirmed using pigs for evaluation of the efficacy of the rFla-Cap protein in this study; furthermore, the capabilities of immune response elicited by the rFla-Cap protein are comparable to the commercial subunit Cap vaccine. Research data [4,14,30,32,38] demonstrated that induction of humoral response alone is not enough to provide full protection for pigs against PCV2 challenge, and cell-mediated immunity, including PCV2-specific IFN-γ response, also promotes the efficacy of the PCV2 vaccines. Additions of some adjuvant in PCV2 subunit vaccines will facilitate to enhancing immune response and promoting protection for pigs from PCV2 infection, because the Cap protein alone-induced IFN-γ response are lower as compared to the whole virus [30]. In the present study, we found that significant upregulation of IFN-γ response, which exceeded the level of IFN-γ response mediated by the commercial Cap subunit vaccine used, was achieved, when administered to pigs with rFla-Cap protein at the high dose. This may be associated with that the binding of flagellin to TLR5 strengthens the activation of cellular response and the uptake of dendritic cells [48,49]. Therefore, it is implicated that the rFla-Cap protein may become an alternatively novel subunit vaccine for control of PCV2 infection due to its good cell-mediated immune response when administered without adjuvant.

Under field conditions, maternally derived antibodies were widely present and diversified in pigs due to the ubiquitous dissemination of PCV2 [50–53]. High maternal PCV2 antibodies might interfere with the development of immunity from vaccination [4,30]. Therefore, an ideal PCV2 vaccine should possess an ability to overcome maternal immunity and confer good protection against infection, even in pigs with PCV2-specific maternal immunity. Research data [28] suggested that vaccination with rCap protein produced in recombinant baculovirus-infected *Trichoplusia ni-larve* when administered with adjuvant was capable of producing high PCV2-specific antibody levels in pigs in the presence of maternal antibodies. Flagellin, which was mainly derived from *Salmonella typhimurium fljb*, possesses a broad range of adjuvant activity. In addition, pre-existing immune response to flagellin does not influence its role as a potential adjuvant for induction of robust responses [54]. However, whether the rFla-Cap protein is capable of conferring good protection for pigs the presence of maternal PCV2-specific antibodies against PCV2 challenge needs to be further determined in the future study.

## Conclusions

In conclusion, the present study here demonstrated that the rFla-Cap protein was capable of eliciting both strong humoral and cell-mediated responses that conferred good protection against PCV2 experimental challenge in pig model, as evidenced by significantly reduced PCV2 viremia, mild lesions, low IHC-positive cells, as well as high levels of PCV2-specific antibodies and high frequencies of IFN-γ-SC, comparable to commercial PCV2 subunit vaccine. However, further experimental studies using commercial farm pigs with maternal PCV2-specific antibodies are required to make firm conclusions regarding good efficacy of the rFla-Cap protein in comparison with commercial PCV2 vaccines.

## Supporting Information

S1 ARRIVE Checklist “The ARRIVE Guidelines Checklist” for reporting animal data. (DOC)
